# Impact of Heat Shock Protein 90 Inhibition on the Proteomic Profile of Lung Adenocarcinoma as Measured by Two-Dimensional Electrophoresis Coupled with Mass Spectrometry

**DOI:** 10.3390/cells8080806

**Published:** 2019-07-31

**Authors:** Ángela Marrugal, Irene Ferrer, Maria Dolores Pastor, Laura Ojeda, Álvaro Quintanal-Villalonga, Amancio Carnero, Sonia Molina-Pinelo, Luis Paz-Ares

**Affiliations:** 1 H12O-CNIO Lung Cancer Clinical Research Unit, Instituto de Investigación Hospital 12 de Octubre & Centro Nacional de Investigaciones Oncológicas (CNIO), 28029 Madrid, Spain; 2 CIBERONC, 28029 Madrid, Spain; 3 Instituto de Biomedicina de Sevilla (IBIS) (HUVR, CSIC, Universidad de Sevilla), 41013 Sevilla, Spain; 4 Program in Molecular Pharmacology, Memorial Sloan Kettering Cancer Center, New York, NY 10065, USA; 5 Medical Oncology Department, Hospital Universitario Doce de Octubre, 28041 Madrid, Spain; 6 Medical School, Universidad Complutense, 28040 Madrid, Spain

**Keywords:** lung cancer, proteomic, chaperones, HSP90 inhibitors

## Abstract

Heat shock protein 90 (HSP90) is an important chaperone in lung adenocarcinoma, with relevant protein drivers such as EGFR (epidermal growth factor receptor) and EML4-ALK (echinoderm microtubule-associated protein-like protein4 fused to anaplastic lymphoma kinase) depending on it for their correct function, therefore HSP90 inhibitors show promise as potential treatments for lung adenocarcinoma. To study responses to its inhibition, HSP90 was pharmacologically interrupted by geldanamycin and resorcinol derivatives or with combined inhibition of HSP90 plus HSP70 in lung adenocarcinoma cell lines. Two-dimensional electrophoresis was performed to identify proteomic profiles associated with inhibition which will help to understand the biological basis for the responses. HSP90 inhibition resulted in altered protein profiles that differed according the treatment condition studied. Results revealed 254 differentially expressed proteins after treatments, among which, eukaryotic translation initiation factor3 subunit I (eIF3i) and citrate synthase demonstrated their potential role as response biomarkers. The differentially expressed proteins also enabled signalling pathways involved in responses to be identified; these included apoptosis, serine-glycine biosynthesis and tricarboxylic acid cycle. The proteomic profiles identified here contribute to an improved understanding of HSP90 inhibition and open possibilities for the detection of potential response biomarkers which will be essential to maximize treatment efficacy in lung adenocarcinoma.

## 1. Introduction

Lung cancer is the leading cause of cancer-related death globally, with a 5-year relative survival rate of only 18% on account of it being commonly diagnosed at advanced stages [1]. There are two major types of lung cancer non-small-cell lung cancer (NSCLC), which accounts for 85% of lung tumors, and small-cell lung cancer (SCLC) accounting for the rest. In turn, NSCLCs are histologically classified according to three subtypes: adenocarcinoma, squamous cell carcinoma and large cell carcinoma [2], with several molecular alterations underlying each histological subtype. This has allowed therapies that target some of these molecular aberrations to be developed [3,4]. While such targeted treatments have achieved improved responses and survival rates, acquired resistance to these treatments is a problem. Moreover, not all NSCLC molecular subtypes have a specific targeted therapy. There is consequently a clear need for novel and broader-spectrum therapies that improve patient responses [5]. Focusing on adenocarcinoma, the main NSCLC subtype (50%), more than half of the cases are driven by recognized oncogenic alterations, such as epidermal growth factor receptor (EGFR), Kirsten rat sarcoma viral oncogene homolog (KRAS), Echinoderm Microtubule-associated protein-like protein 4 fused to anaplastic lymphoma kinase (EML4-ALK), mesenchymal-epithelial transition (MET) factor, serine/threonine-protein kinase B-Raf (BRAF) or human epidermal growth factor 2 (HER2/ErbB2/neu) [6,7]. As most of these proteins are clients of 90kDa heat shock protein (HSP90) [8], an elevated expression of HSP90 has consequently been correlated with a poorer clinical prognosis [9,10] as well as with resistance to chemo- and radiotherapy [11,12,13,14,15].

HSP90 is one of the most abundant and evolutionarily conserved molecular chaperones. Besides representing 1–2% of all cellular proteins, this chaperone can increase its expression up to 10-fold in response to physiological stress [16]. Dominant isoforms are the constitutively expressed HSP90β and the stress inducible HSP90α which can be found in the cytoplasm, nucleus or even on the cell surface and extracellular space (for HSP90α) [17]. Both isoforms are collectively named HSP90 unless specified, and play a critical role in the maturation, stabilization and regulation of so-called “client” proteins via an ATP-driven chaperone cycle regulated by co-chaperones such as HSP70 and p23 [18]. Many of the approximately 300 client proteins (https://www.picard.ch/downloads/Hsp90interactors.pdf) play key roles in oncogenic signalling and in different hallmarks of cancer such as proliferation, evasion of apoptosis, immortalization, angiogenesis, invasion and metastasis. Due to of their strong dependence on HSP90, inhibition of the latter leads to ubiquitin-mediated proteasomal degradation of client proteins concluding with the downregulation of different oncogenic signalling pathways [19]. Since EGFR [20], BRAF [21], ERBB2 [22], MET [23,24] and the EML4-ALK translocation product [8] are clients of HSP90, acting as oncodrivers in different clinico-pathological subsets of lung adenocarcinoma, degradation of these oncoproteins through HSP90 inhibition leads to loss of tumor-cell viability [25,26,27,28]. Promising results have been shown in different clinical studies, especially in malignancies that possess an HSP90 client as an oncodriver [29,30,31]. However, as not all lung adenocarcinomas respond equally to HSP90 inhibitors [32,33], a better understanding of the cellular consequences of HSP90 inhibition will therefore be key to improve clinical outcomes in this tumor type.

Proteomic methods have been used widely to identify protein network alterations that may be linked to drugs used to treat lung cancer in terms of sensitivity and resistance [34,35,36,37]. Specifically, two-dimensional gel electrophoresis has been employed to analyze protein expression profiles and identify novel diagnostic, prognostic or predictive biomarkers in these tumors [38,39,40,41,42,43,44]. Due to the complex interactome of HSP90 and the enormous number of cellular processes in which it is involved, the use of proteomic tools for the study of this chaperone and its clients is a common approach [17,45,46,47]. However, additional information is needed to dissect responses to HSP90 inhibitors in lung cancer. For this reason, we analyzed proteome modulation by HSP90 inhibitors in different lung adenocarcinoma cell lines using two-dimensional electrophoresis coupled with tandem mass spectrometry. Comparisons of proteomic expression profiles in drug-treated and untreated cells showed remarkable changes in both the number and expression levels of many proteins. Differentially expressed proteins could help in the identification of plausible biomarkers and elucidate cellular processes involved in responses arising from the inhibition of HSP90 in these tumors.

## 2. Materials and Methods

### 2.1. Cell Cultures

The human lung adenocarcinoma cell lines HCC827 (CRL-2868), A549 (CCL-185) and H1437 (CRL-5872) were obtained from American Type Culture Collection (ATCC), and the H3122 cell line was kindly provided by Dr. Koivunen. Cell lines were propagated in RPMI-1640 medium (Sigma-Aldrich, Saint Louis, MO, USA), with the exception of A549 which was cultured in DMEM (Sigma-Aldrich). All cell lines were supplemented with 10% (*v/v*) fetal bovine serum (FBS, TICO Europe, Amstelveen, The Netherlands), 1% (*v/v*) antibiotic-antimycotic solution (Sigma-Aldrich) and 1% (*v/v*) glutamine. Cells were maintained at 37 °C in a humidified incubator in a 5% CO_2_ and 95% air atmosphere. Cells were authenticated and regularly checked for mycoplasma.

### 2.2. siRNA Transfection

Transient silencing of HSP90 was achieved through small interfering RNAs (siRNAs). For HSP90α and HSP90β silencing, siRNAs were purchased from Origene (Rockville, MD USA) (SR302262 and SR302264). In turn, control cells were transfected with scrambled siRNA (SR30002). LipofectamineTM RNAiMAX (Invitrogen, Carlsbad, CA, USA), a cationic lipid transfection reagent, was used to transfect all cell lines according to the manufacturer’s instructions. Simultaneous transfections were performed to silence both genes in the same cell line.

### 2.3. Drug Treatment

The HSP90 inhibitors used in this study included analogues of geldanamycin: tanespimycin (17-N-allylamino-17-demethoxygeldanamycin, 17-AAG) (Selleckchem, Munich, Germany) and retaspimycin hydrochloride ((4E,6Z,8S,9S,10E,12S,13R,14S,16R)-19-(allylamino)-13,20,22-trihydroxy-8,14-dimethoxy-4,10,12,16-tetramethyl-3-oxo-2-azabicyclo[1 6.3.1]docosa-1(22),4,6,10,18,20-hexaen-9-yl carbamate, IPI-504) (Eurodiagnóstico, Madrid, Spain) and radicicol derivatives: ganetespib (3-(2,4-Dihydroxy-5-isopropylphenyl)-4-(1-methylindol-5-yl)-5-hydroxy-4H-1,2,4-triazole, STA-9090) and luminespib (5-(2,4-dihydroxy-5-isopropylphenyl)-N-ethyl-4-(4-(morpholinomethyl)phenyl)isoxazole-3-carboxamide, AUY-922) (Selleckchem). VER-155008 (5’-O-[(4-Cyanophenyl)methyl]-8-[[(3,4-dichlorophenyl)methyl]amino]-adenosine) (ApexBio, Houston, TX, USA) was used as an HSP70 inhibitor. The chemical structure of all inhibitors used can be accessed on the website https://pubchem.ncbi.nlm.nih.gov/. All drugs were dissolved in dimethyl sulfoxide (DMSO) as stock solutions according to manufacturers’ instructions. Cells were treated for 96 h with the different inhibitors in concentrations ranging from 0.33 nM to 20 uM to calculate half-maximal inhibitory concentrations (IC50). Thereafter, the concentration for each drug at which growth is reduced to 80% (IC80) was calculated and applied to cell lines maintained in complete growth medium. Protein extracts were collected at different times (10, 24 and/or 48 h) according to subsequent techniques to be used.

### 2.4. Immunoblotting

Total protein extracts from the studied cell lines were isolated with RIPA buffer (Sigma-Aldrich) containing a protease inhibitor cocktail (cOmpleteTM Mini EDTA-free, Roche, Basel, Switzerland) and a phosphatase inhibitor cocktail (PhosSTOP EASYpack, Roche). A standard western blot protocol was employed with a miniProtean electrophoretic system (BioRad, Berkeley, CA, USA) and a wet electroblotting system (BioRad). Primary antibodies against HSP90α (ab79849, Abcam, Cambridge, UK), HSP90β (ab53497, Abcam), GRP94 (ab18055, Abcam), HSP70 (ab45133, Abcam), HSP27 (#2402, Cell Signaling, Danvers, MA, USA) EGFR (#4267, Cell Signaling), pEGFR (#2234, Cell Signaling), ALK (#3633, Cell Signaling), eIF3i (ab40745, Abcam), citrate synthase (#14309, Cell Signaling), α-Tubulin (T9026, Sigma-Aldrich) or β-actin (A5316, Sigma-Aldrich) were used. Antibody-bound proteins were detected by enhanced chemiluminescence (Clarity Western ECL Blotting Substrates (BioRad)) using the ChemiDoc system (BioRad) after incubation with horseradish peroxidase-conjugated anti-mouse secondary antibody (#7076, Cell Signaling) or anti-rabbit secondary antibody (#7074, Cell Signaling). The ratios between signals from proteins of interest and α-tubulin or β-actin were calculated to determine the relative protein expression values. No grouping of gels/blots cropped from different parts of the same gel, or from different gels, fields or exposures was performed.

### 2.5. Proteomic Sample Preparation

Cells were seeded into 10 cm diameter dishes with the relevant culture medium at the appropriate density to reach 80 percent confluence 24 h post-seeding. On the following day, cells were treated with the different HSP90 inhibitors or the HSP90 (17-AAG) plus HSP70 (VER-155008) inhibitors at its IC80 while the negative control was treated with the same amount of vehicle DMSO used in the inhibited cells. After 24 h, cells were collected, precipitated, washed with ice-cold PBS and finally each pellet was shock-frozen in liquid nitrogen and stored at −80 °C until further use. Before use, pellets were thawed on ice and then cells were resuspended in HEN buffer (50 mM HEPES (Sigma-Aldrich), 5 mM ethylenediaminetetraacetic acid (EDTA) (Invitrogen Ambion), 250 mM sodium chloride (NaCl) (Panreac, Barcelona, Spain), 5 mM dithiothreitol (DTT) (GE Healthcare, Chicago, IL, USA), 1 mM sodium orthovanadate (Sigma-Aldrich), 0.2% IGEPAL (Sigma-Aldrich) and 5 µL of protease inhibitor cocktail (Sigma-Aldrich)) and lysed for 1 h on ice with intermittent vortexing.

Protein samples from each cell line and condition were pre-treated with the 2-D Clean Up Kit (GE Healthcare) and the final pellets were resuspended in 2-DE lysis buffer (8 M urea (GE Healthcare), 4% CHAPS (GE Healthcare), 2% IPG buffer pH 3-11 nonlinear (NL) (GE Healthcare) and 40 mM DTT(GE Healthcare)). Protein samples were maintained overnight at 4 °C with gentle rotation to ensure their complete resolubilization. Protein concentrations in the 2-DE samples were determined by the 2-D Quant-Kit (GE Healthcare).

### 2.6. Two-Dimensional Electrophoresis and Gel Image Analysis

In the experiment, 240 µg of total protein from each individual sample were diluted to up to 250 µL with DeStreak rehydration solution (GE Healthcare) and 0.5% 3–11 NL pH IPG buffer (GE Healthcare). Each mixture was loaded onto 13 cm IGP DryStrips (GE Healthcare) with a 3–10 NL pH gradient. The first-dimension separation—isoelectric focusing (IEF) —was then carried out on an Ettan IPGphor II system (GE Healthcare). IPG DryStrips were equilibrated in a reducing agent followed by an alkylating agent. The second dimension was performed by placing the strips on 8–16% CriterionTM TGX stain-free acrylamide gels (Bio-Rad) to allow protein separation by electrophoresis in a Criterion DodecaTM cell (Bio-Rad). The analytical gels were visualized with Typhoon 9400 (GE Healthcare) after SYPRO^®^ (Bio-Rad) staining. The digitalized 2-DE gel images were studied (protein spot detection, spot matching and semi-quantitative statistical analysis) using PDQuest software (Bio-Rad). For each studied condition, three different gel images were analyzed and a corresponding reference synthetic image was obtained. To improve accuracy, detected spots and spot matches after in silico matching, were manually edited.

### 2.7. MALDI-TOF/TOF Mass Spectrometry Analysis

Spots that were present in only one of the conditions or displayed quantitative abundance changes of more than 2-fold were selected for identification by MALDI-TOF/TOF. Protein spots of interest were picked from the stained gel using the Investigator ProPic robotic workstation (Genomic Solution, Huntingdon, UK) and were then washed and digested. The samples were mixed with a matrix solution CCA (α-cyano-4-hydroxycinnamic acid), spotted on a MALDI plate (Applied Biosystems, Foster City, CA, USA) and allowed to air-dry. To obtain a peptide mass fingerprint (PMF), lists of peak intensities and mass-to-charge (*m/z*) values were analyzed with a 4800 Proteomics Analyzer MALDI-TOF/TOF Mass Spectrometer (Applied Biosystems).

### 2.8. Protein Identification

High-resolution tandem mass spectra MS + (MS/MS) data, peptide matching and protein identification were determined with web-based MASCOT software (http://www.matrixscience.com/) using SWISS-PROT as the protein sequence database with a mass tolerance of ± 50 ppm. Candidate proteins were selected from each spot, using the number of matched peptides, sequence coverage, molecular mass and isoelectric point, among other variables, as criteria for accepting the identification.

### 2.9. Protein Functional Analysis

The gene ontology enrichment analysis of identified proteins was performed using the PANTHER (Protein ANalysis THrough Evolutionary Relationships) online database (http://pantherdb.org/) [48,49]. Pathway analyses were carried out for differentially expressed proteins from the cell lines studied in response to different inhibitors. This database was also used to categorize identified proteins according to their biological processes and molecular functions. Only results whose adjusted p-value was lower than 0.05 were considered statistically significant and used in the study.

## 3. Results

### 3.1. Characterization of Lung Adenocarcinoma Cell Lines

**Figure 1:**
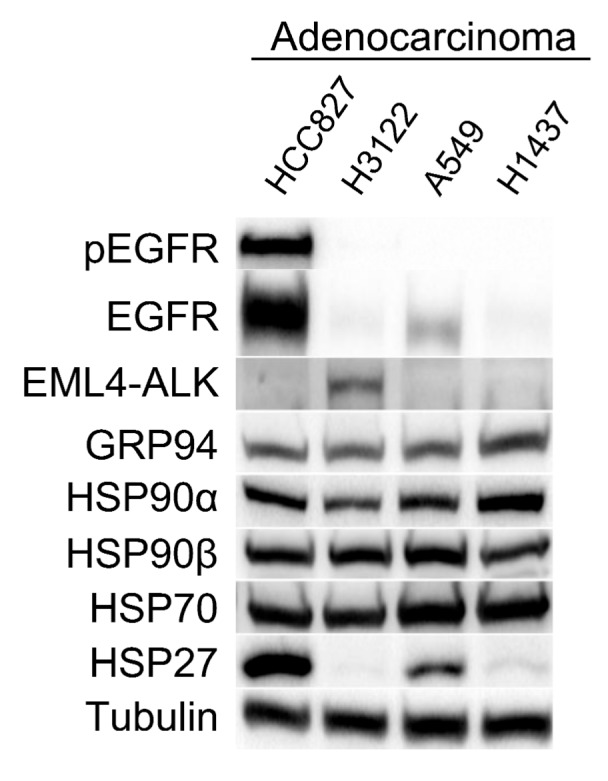
Characterization of a panel of adenocarcinoma cell lines. Western blot analysis of adenocarcinoma cell lines whose oncodrivers are either directly (epidermal growth factor receptor (EGFR) in HCC827 and EML4-ALK in H3122 cell lines) or indirectly Kirsten rat sarcoma (KRAS) viral oncogene homolog) in the A549 cell line) related to HSP90, as well as an EGFR, anaplastic lymphoma kinase (ALK) and KRAS wild-type cell line (H1437). This technique was employed to study protein expression levels of constitutive and inducible HSP90, other related heat shock proteins (HSPs) (GRP94, HSP70 and HSP27) as well as the HSP90 clients EGFR, its phosphorylated form pEGFR, and EML4-ALK. For Western blots, a representative image is shown. Densitometry analysis may be found in Figure S1.

Lung adenocarcinoma cell lines from different backgrounds were characterized based on protein expression levels measured for HSP90, other related HSPs and HSP90 client proteins such as EGFR and EML4-ALK (Figure 1). The EGFR-mutant cell line (HCC827) showed highest level of EGFR expression (followed by the KRAS-mutant A549 cells) and was the only cell line showing EGFR activation. The presence of EML4-ALK fusion protein was detected exclusively in the H3122 cell line. Focusing on HSPs, three of the four cell lines studied showed similar levels of cytosolic HSP90 (α and β) protein expression, while the H1437 cell line (triple-negative; EGFR, ALK and KRAS wild-type) exhibited a higher expression of HSP90α and a lower expression of HSP90β than the other cell lines studied. Similarly, GRP94 (the endoplasmic reticulum HSP90 isoform) expression was higher in the H1437 cell line than the other cell lines. On the other hand, protein levels of HSP70 were similar in all cell lines although slightly higher in the A549 and H1437 cell lines. Furthermore, the HCC827 and A549 cell lines showed high HSP27 protein expression levels compared to almost undetectable levels in the other cell lines.

### 3.2. Effectiveness of HSP90 Inhibitors in Lung Adenocarcinoma Cell Lines

Cells were treated with different doses of HSP90 (17-AAG, IPI-504, STA-9090 and AUY-922) and HSP70 (VER-155008) inhibitors with a view to calculating IC50 values and examining the efficacy of each drug in each cell line (Table 1).

**Figure 2:**
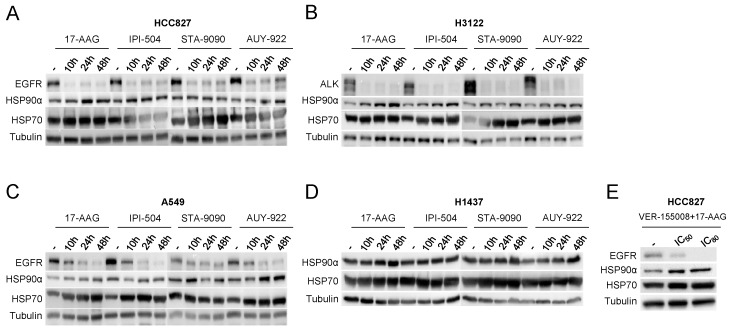
Analysis of sensitivity of adenocarcinoma cell lines to HSP90 inhibitors. Western blot determination of the effect of 17-AAG, IPI-504, STA-9090 and AUY-922 inhibitors on adenocarcinoma cell lines characterized by (**A**) mutated EGFR, (**B**) EML4-ALK translocation, (**C**) mutated KRAS and (**D**) EGFR, ALK and KRAS wild-type. (**E**) Study of combined treatment of HSP70 and HSP90 inhibitors in the HCC827 cell line. HSP90α and HSP70 expression was determined in each of the cell lines along with the HSP90 clients EGFR and EML4-ALK. For Western blots, a representative image is shown. Densitometry analysis may be found in Figure S2.

The studied cell lines (especially the cell line harboring EML4-ALK translocation, H3122) were more sensitive to HSP90 inhibitors than to the HSP70 inhibitor used. In relation to HSP90 inhibition, IC50 values ranged from 2814 to 30,733 µM, with the radicicol derivatives (STA-9090 and AUY-922) generally having lower IC50 values particularly in the EGFR-mutant and EML4-ALK translocated cell lines. Subsequently, western blot analyses were used to evaluate responses to HSP90 inhibition at different time points based on IC80 values (Figure 2). We identified that HSP90 inhibitors induced compensatory expression of HSP70 and HSP90, along with significant reductions in the HSP90 clients EGFR and EML4-ALK since the 10 h time-point in analyzed treatments. Moreover, EGFR degradation was faster and more pronounced in the EGFR-mutant cell line HCC827 than in the KRAS-mutant cell line A549 despite the high initial expression of EGFR. It is also worth noting that HSP90 inhibition in the H3122 cell line dramatically inhibited expression of its oncodriver EML4-ALK.

### 3.3. Effects of HSP90 Inhibition on Protein Expression Patterns in Adenocarcinoma Cell Lines

To identify proteins correlated with HSP90 inhibition, we used 2-DE to analyze the proteome from each cell line treated with the four selected HSP90 inhibitors, the HSP90 (17-AAG) plus HSP70 (VER-155008) inhibitors combination, or vehicle treatment for 24 h. To eliminate gel to gel variation, isoelectric focusing and the second dimension of all samples for each cell line were performed at the one time in a single assay. Different proteome profiles of treated and control groups in the studied cell lines could be visualized following image analysis by PDQuest 2-D software. Representative 2-DE gels for inhibited and untreated HCC827, H3122, A549 and H1437 cells are shown in Figures S3–S7 where the two most over- and down-expressed protein spots for each condition and cell line are given. An average of 473, 201, 257 and 263 protein spots were detected, quantified, normalized and inter-gel-matched in the EGFR, EML4-ALK, KRAS, and triple-negative cell lines, respectively (Table 2).

Protein spots included in the analyses were those with significant quantitative differences of at least 2-fold expression variation between treatments and control or those qualitative spots present only in some of the treatments or the control. The results, after mass spectrometry analysis, of identified protein variations after treatments are shown in Table 3 and Tables S1–S5.

The EGFR (HCC827) cell line showed the highest number of deregulated proteins after treatment with the different HSP90 inhibitors studied without major differences between treatments. The triple-negative cell line (H1437) had a higher number of differentially expressed proteins following treatment with geldanamycin derivatives whereas more deregulated proteins were detected in the EML4-ALK (H3122) and KRAS (A549) cell lines after treatment with radicicol derivatives. The KRAS cell line was the only line where the number of down-expressed proteins was higher than that of overexpressed proteins, concretely after treatment with radicicol derivatives.

Regarding differentially expressed proteins in the EGFR cell line, HSP70, enolase and mutL-like 1 (MLH1) protein were among the most overexpressed proteins, while HSP90AA1 (HSP90α), sumo3 and HSC71 were the most down-expressed. In the EML4-ALK cell line, anterior gradient protein 2 homolog (AGR2) was the most overexpressed protein while eukaryotic translation initiation factor 3 subunit I (eIF3i) was the most down-expressed protein after inhibition. MHC class I antigen, citrate synthase (CS), transketolase and elongation factor 1-alpha were some of the most overexpressed proteins in the KRAS cell line, while pyruvate kinase and ATP synthase subunit beta were among the most down-expressed. Lastly, in the triple-negative cell line, transketolase, mitochondrial aldehyde dehydrogenase 2 variant (ALDH2) and phenylalanine tRNA ligase beta subunit were among the most overexpressed proteins, whereas 3-hydroxyacyl-CoA dehydrogenase type-2, alternative protein fibroblast growth factor 1 (FGF1) and creatine kinase U-type were commonly detected as down-expressed after treatments.

Concerning HSP90 inhibitors, the radicicol derivative STA-9090 was the treatment in response to which more proteins were deregulated and the only one that showed a higher number of down-expressed than overexpressed proteins. On the other hand, the geldanamycin derivative IPI-504 showed the second highest dysregulation of the HSP90 inhibitors. It was also the treatment which produced the largest number of overexpressed proteins, which was mainly due to the response of the A549 and H1437 cell lines exposed to this inhibitor. Finally, the HSP90 (17-AAG) plus HSP70 (VER-155008) inhibitor combination led to greater protein dysregulation than that seen with the HSP90 inhibitors studied alone. Like under HSP90 inhibition, the highest number of deregulated proteins was detected in the EGFR cell line. Besides, this along with the KRAS cell line were the only cell lines where the number of down-expressed proteins was greater that than of overexpressed proteins in response to the combined inhibition treatment.

Following an in-depth analysis of the geldanamycin-derivative inhibition, we identified 65 differentially expressed proteins in the 17-AAG treated cell lines, four of which were deregulated in several of the studied cell lines. eIF3 subunit I was down-expressed in the HCC827 (EGFR-mutated) and H3122 (EML4-ALK rearrangement) cell lines. MHC class 1 antigen was down-expressed after treatment of HCC827 cells but overexpressed in A549 (KRAS-mutated) cells. Ras-related protein Rab-37 and transketolase were also identified in the A549 as well as in the H1437 (triple-negative; EGFR, ALK and KRAS wild-type) cell lines. Rab-37 was overexpressed after treatment of A549 cells but down-expressed in H1437 cells. Transketolase on the other hand was overexpressed in both cell lines. In the case of the IPI-504 treatment, 79 proteins were identified. Five of them were deregulated in two or more of the cell lines tested. HSP90α was down-expressed in the EGFR-mutated cell line (HCC827) and overexpressed in the ALK translocation (H3122) cell line. HSP70 protein in its 1A and 1B variants was overexpressed in HCC827 and A549 cell lines in response to treatment. Transketolase was detected again, this time in three different cell lines, where its expression was decreased in H3122 and increased in A549 and H1437 cells after inhibition with IPI-504. Finally, Heat shock cognate 71 kDa protein (HSC71), also known as heat shock 70 kDa protein 8 (HSPA8), was down-expressed in HCC827 and overexpressed in H1437 cells.

With respect to the radicicol derivatives used, inhibition with STA-9090 led to 107 different proteins being identified, four of which were found in two or more cell lines. HSP90α as well as the mitochondrial 60kDa heat shock protein, were down-expressed in HCC827 cells but overexpressed in H3122 cells. Rab-37 was again identified as being down-expressed following treatment of the HCC827 and A549 cell lines. Finally, the MHC class II antigen was overexpressed in H3122 cells and down-expressed in A549 cells. On the other hand, sixty-nine differentially expressed proteins were detected after cells were treated with AUY-922. Only two proteins were identified in more than one cell line. HSP70 was overexpressed in HCC827 and A549 cells while transketolase was overexpressed in A549 and H1437 cells.

Combination of the HSP90 inhibitor 17-AAG plus the HSP70 inhibitor VER-155008 gave rise to 143 deregulated proteins, 9 of which were common to two cell lines. Six proteins were deregulated in HCC827 and A549 cells. Retinoic acid receptor RXR-beta, lipoamide acyltransferase component of branched-chain alpha-keto acid dehydrogenase complex mitochondrial and cysteine and glycine-rich protein 2 were down-expressed while HSP70 1A was overexpressed in both cell lines. SAM and SH3 domain-containing protein 1 were overexpressed in HCC827 and down-expressed in A549 cells. In contrast, MHC class I antigen expression was decreased in HCC827 cells and increased in A549 cells. Heat shock 70 kDa protein 6 was overexpressed in the H3122 and A549 cell lines, while 60S acidic ribosomal protein P0 was one of the more highly expressed proteins following treatment of A549 and H1437 cells. The double inhibition treatment resulted in transketolase overexpression in HCC827 and H1437 cells.

With the purpose of searching a common protein signature after HSP90 inhibition and taking together results for the most deregulated proteins and those detected in two or more cell lines for the same inhibitor, six shared proteins (HSP70, HSP90α, HSC71, eIF3i, MHC class 1 antigen and transketolase) were identified to be involved in the response to HSP90 inhibitors. It is important to note that adenocarcinoma is a molecularly diverse entity, in which many different molecular alterations have been identified as drivers of different subsets of patients in this context, each of them showing different prognosis and response to therapy. This fact makes each cell line harboring a different molecular driver unique. Nevertheless, a protein dysregulation pattern common for all cell lines tested was identified in this study, where 90% of the dysregulated proteins were found among the first ten proteins with average ratios greater than 5, which indicates the strength of our results. It is also remarkable that not only the expected HSP70, but also eIF3i, were detected unexpressed and down-expressed, respectively, in different cell lines.

### 3.4. Functional Annotation Analysis of Differentially Expressed Proteins

**Figure 3:**
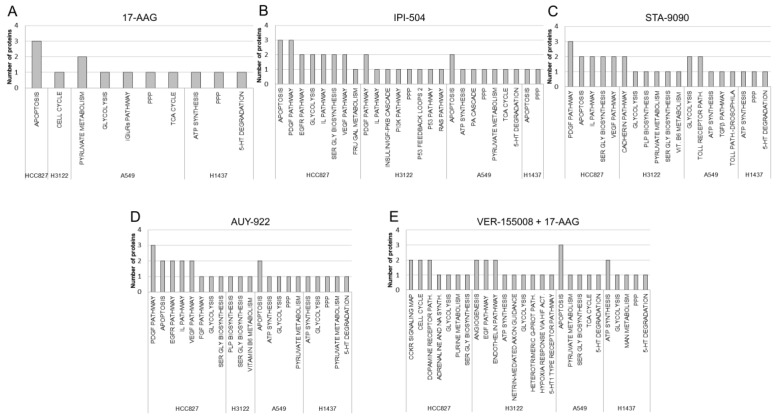
Canonical pathway analysis of proteins identified by two-dimensional electrophoresis and mass spectrometry (2-DE/MS) in response to HSP90 inhibition. Functional analysis of the differentially expressed proteins in response to (**A**) 17-AAG, (**B**) IPI-504, (**C**) STA-9090, (**D**) AUY-922 and (**E**) VER-155008 + 17-AAG inhibitors in the adenocarcinoma cell lines HCC827, H3122, A549 and H1437. Enriched pathways were determined from the PANTHER database. The number of proteins involved in each identified pathway is represented on the y axis. iGluRs pathway = ionotropic glutamate receptor pathway, PPP= pentose phosphate pathway, TCA cycle = tricarboxylic acid cycle, 5-HT degradation = 5-hydroxytryptamine or serotonin degradation, PDGF pathway = platelet-derived growth factor signaling pathway, EGFR pathway = epidermal growth factor receptor pathway, IL pathway = interleukin signaling pathway, SER GLY biosynthesis = serine and glycine biosynthesis, VEGF pathway = vascular endothelial growth factor signaling pathway, FRU GAL metabolism = fructose and galactose metabolism, insulin/IGF-PKB cascade = insulin/ insulin-like growth factor pathway-protein kinase B signaling cascade, PI3K pathway = phosphoinositide-3 kinase pathway, PA cascade = plasminogen activating cascade, PLP biosynthesis = pyridoxal-5-phosphate biosynthesis, TGFβ pathway = transforming growth factor beta signaling pathway, FGF pathway = Fibroblast Growth Factor signaling pathway, adrenaline and NA synth. = adrenaline and noradrenaline biosynthesis, EGF pathway = epidermal growth factor pathway, heterotrimeric G-protein signaling pathway = heterotrimeric G-protein signalling pathway Gi alpha- and Gs alpha-mediated pathway, 5-HT1 type receptor pathway = serotonin 1A receptor-mediated signalling pathway, MAN metabolism = mannose metabolism.

Proteins that were differentially expressed in response to the different HSP inhibitors were analyzed using PANTHER software for mapping of the molecular pathways that were possibly involved (Figure 3). Results showed that the deregulated proteins identified in response to 17-AAG inhibition participated in nine pathways, including the pentose phosphate pathway (PPP), which was the only pathway enriched in two different cell lines (A549 (KRAS) and H1437 (TN)) (Figure 3A). In the case of the other geldanamycin-derivative inhibitor, IPI-504 19, enrichment pathways were identified that included the platelet-derived growth factor (PDGF) and interleukin pathways which were shared by the EGFR (HCC827) and the EML4-ALK (H3122) cell lines. Two other pathways also coincided, consisting of apoptosis in HCC827, A549 and H1437 cells, and PPP in H3122, A549 and H1437 cells (Figure 3B). On the other hand, for radicicol derivatives, the set of STA-9090-associated proteins were assigned to 16 pathways, three of which were shared by different cell lines: serine glycine synthesis (detected in the EGFR and EML4-ALK cell lines), deregulated glycolysis (in the EML4-ALK and KRAS cell lines) and ATP synthesis (in the KRAS and triple-negative cell lines) (Figure 3C). AUY-922 was the HSP90 inhibitor with more shared pathways among the cell lines studied. These included serine glycine synthesis (again detected in the EGFR and the EML4-ALK cell lines), glycolysis (enriched in the EGFR, KRAS and triple-negative cell lines), and deregulated apoptosis (in the EGFR and KRAS cell lines). The A549 (KRAS) and H1437 (TN) cell lines also showed enrichment in ATP synthesis, PPP and pyruvate metabolism (Figure 3D).

It is noteworthy that the largest number of pathways was identified when a HSP90 inhibitor was used in combination with the HSP70 inhibitor (VER155008). Four of 21 pathways were shared by cell lines. Glycolysis was detected in the EGFR, EML4-ALK and triple-negative cell lines, while serine glycine synthesis was found in the EGFR and KRAS cell lines. Moreover, ATP synthesis was enriched in the EML4-ALK and triple-negative cell lines such as 5-hydroxytryptamine (5-HT) degradation was found in the KRAS and triple-negative cell lines (Figure 3E).

### 3.5. Protein Expression Profiles and Signalling Pathways Associated with the HSP90 Inhibitor Family in Lung Adenocarcinoma Cell Lines

**Figure 4:**
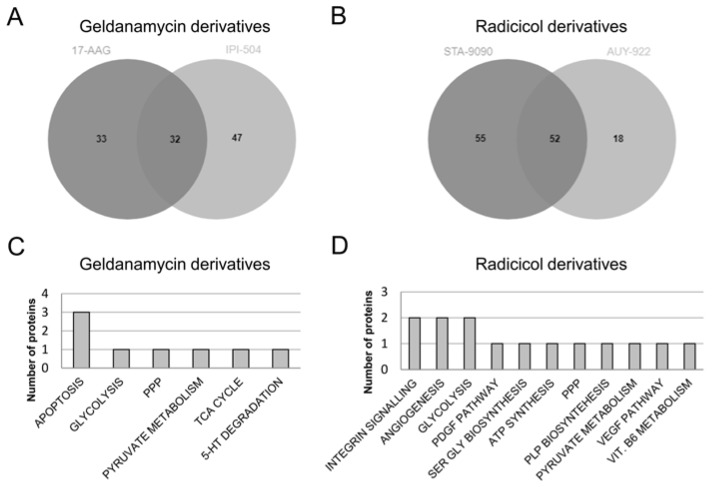
Study of cell line response to HSP90 inhibitors according to inhibitor family. Venn diagrams showing overlap of differentially expressed proteins as well as those specific to inhibition with (**A**) geldanamycin or (**B**) radicicol derivatives. The analysis of shared proteins following (**C**) geldanamycin or (**D**) radicicol treatments was performed with PANTHER software which identified deregulated pathways associated with each HSP90 inhibitor family. The number of proteins involved in each identified pathway is represented on the y axis. PPP= pentose phosphate pathway, TCA cycle = tricarboxylic acid cycle, 5-HT degradation = 5-hydroxytryptamine or serotonin degradation, PDGF pathway = platelet-derived growth factor signaling pathway, SER GLY biosynthesis = serine and glycine biosynthesis, PLP biosynthesis = pyridoxal-5-phosphate biosynthesis, VEGF pathway = vascular endothelial growth factor signaling pathway.

From the deregulated proteins under HSP90 inhibition in the four studied cell lines, Venn diagrams were constructed to illustrate the overlap of altered proteins within each HSP90 inhibitor family. Among the differentially expressed proteins after treatment with the geldanamycin derivatives, 33 were specific for 17-AAG and 47 for IPI-504, with 32 proteins (Table S6) common to both inhibitors (Figure 4A). Among the proteins common to both inhibitors, the overexpression of HSP70, CS, AGR2, enolase and sumo3 are of note. On the other hand, only V-ATPase 116 kDa isoform a1, anamorsin, calcium-independent phospholipase A2-gamma (iPLA2-2) and 3-hydroxyacyl-CoA dehydrogenase type-2 were exclusively down-expressed under geldanamycin derivatives. Four proteins (HSC71, MHC class I antigen, HSP90α and transketolase) showed different expression patterns depending on the cell line under consideration.

The radicicol derivatives group shared 52 deregulated proteins (Table S7). Fifty-five proteins were exclusively related to STA-9090 treatment while 18 were exclusive to AUY-922 inhibition (Figure 4B). Among the 30 shared proteins that were overexpressed, HSP70, AGR2 and enolase were again present in addition to new ones such as transketolase, vinculin or radixin. A smaller number of proteins were down-expressed such as V-ATPase 116 kDa isoform a1, iPLA2-2, 3-hydroxyacyl-CoA dehydrogenase type-2, ATP synthase subunit beta, HSC71, eIF3i and sumo3. MHC class I antigen, HSP90α, pyruvate kinase (PKM) and glucose-6-phosphate 1-dehydrogenase (G6PD) showed different protein expression patterns according to the cell lines in question.

We identified that proteins that were deregulated following treatment with geldanamycin derivatives were involved in six pathways, while those detected after radicicol derivative treatments participated in 11 pathways. Three pathways (apoptosis, TCA cycle and 5-HT degradation) were exclusively enriched in response to treatment with geldanamycin derivatives (Figure 4C). The differentially expressed proteins related to these pathways were the different isoforms of HSP70 and HSC71 (for apoptosis), CS (for TCA cycle) and aldehyde dehydrogenase (for 5-HT degradation). In turn, the integrin signalling pathway, angiogenesis, the PDGF pathway, serine glycine biosynthesis, ATP synthesis, pyridoxal-5-phosphate (PLP) biosynthesis, the VEGF pathway and vitamin B6 metabolism were specific to radicicol derivative treatment (Figure 4D). In this case, the proteins related to the enriched pathways (in parentheses) were BRAF and vinculin (integrin signalling pathway), BRAF and FGF1 (angiogenesis), BRAF (PDGF pathway), phosphoserine aminotransferase (serine glycine biosynthesis), ATP synthase subunit α and β (ATP synthesis), phosphoserine aminotransferase (PLP biosynthesis), BRAF (VEGF pathway) and phosphoserine aminotransferase (vitamin B6 metabolism).

Only three signalling pathways (PPP, glycolysis, and pyruvate metabolism) were detected across the two HSP90 inhibitor families. Transketolase was the protein whose dysregulation was involved in the enrichment of PPP in both inhibitor families. Enolase was the common protein related to glycolysis dysregulation, but PKM also took part in inhibition by radicicol family derivatives. The final common pathway, pyruvate metabolism, was related to CS for inhibition by geldanamycin derivatives and to PKM for radicicol derivatives.

### 3.6. Validation of the Differential Expression of Proteins Involved in the Response to Chaperone Inhibitors

**Figure 5:**
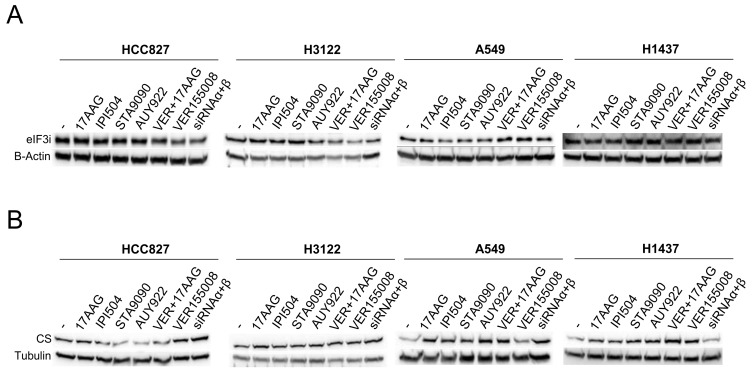
Western blotting validation of proteins identified as differentially expressed in the 2-DE/MS analysis. Protein lysates from HCC827, H3122, A549 and H1437 cells without treatment or inhibited with 17-AAG, IPI-504, STA-9090, AUY-922 VER-155008 + 17AAG and VER-155008 as well as the genetic silencing of HSP90 were immunoblotted. Expression study of (**A**) eIF3i and (**B**) CS after HSP90 inhibition as well as its loading control are shown. eIF3i = eukaryotic translation initiation factor 3 subunit I, CS = citrate synthase. For Western blots, a representative image is shown. Densitometry analysis may be found in Figure S8.

Two proteins (eIF3i and CS) were evaluated by western blotting owing to their important changes in expression in different cell lines in response to inhibitors as well as to their relationship with the deregulated pathways. The first of these, eIF3i, was the most down-expressed protein in response to treatment with 17-AAG, STA-9090, and VER-155008 + 17-AAG, and the second one under AUY-922 in the EML4-ALK cell line H3122. The down-expression of this important initiation factor was also detected in HCC827, the EGFR cell line, under the inhibitor 17-AAG. Western blotting of eIF3i and the corresponding internal control of the four studied cell lines are shown in Figure 5A. Since this technique is not as sensitive as 2-DE, the expression trend was observed more clearly in genetic silencing of HSP90 (α and β) than under drug inhibition of HSP90 in the EGFR and EML4-ALK cell lines. At the same time, these cell lines presented a noticeable eIF3i degradation after treatment with VER-155008, the HSP70 inhibitor, alone or in combination with 17-AAG. In contrast, the triple-negative cell line (H1437), showed an increased expression after genetic silencing of HSP90 and most of the studied conditions. In fact, H1437 cells showed an exclusive, small decreased expression of eIF3i after inhibition with 17-AGG in monotherapy or in conjunction with VER-155008. Finally, the KRAS cell line showed an intermediate expression pattern with increased expression of eIF3i after HSP70 inhibitor in conjunction with 17-AAG, as well as a decreased expression of this protein under genetic silencing or inhibition of HSP90.

We also validated CS, which was differentially expressed across cell lines. This protein is an enzyme of the TCA cycle whose regulation is related to ATP synthesis and other metabolic pathways such as glycolysis, which were detected as deregulated pathways after HSP90 inhibition in the enrichment analysis. CS showed the largest increase of expression in the A549 cell line after treatments, confirming the 2-DE results, where approximately 7-fold expression changes were detected after 17-AAG, IPI-504 and VER-155008 + 17-AAG treatment. It should be pointed out that the EGFR cell line HCC827 was the only line where a decrease in expression of this protein after treatments was detected (Figure 5B).

## 4. Discussion

In this study we used 2-DE coupled with mass spectrometry to analyze differences that take place in the proteome of lung adenocarcinoma cell lines in response to HSP90 inhibition. For each lung adenocarcinoma cell line studied, we identified clear alterations in the protein profiles in treated compared to untreated control cell lines. These differences were notable for individual HSP90 inhibitors and in combination with an HSP70 inhibitor, with responses always being more effective for radicicol derivative treatments given the lower IC50 values and higher proteomic dysregulation in the studied cell lines treated with these inhibitors.

Treatment with HSP90 inhibitors of lung adenocarcinoma cell lines, as expected, showed remarkable changes in the homeostasis of HSPs, leading to deregulation of HSP90, HSP70, HSP60, HSC71, HSP70 protein 6 (HSPA6), and mitochondrial HSP70 (mitHSP70). It has been reported that HSP90 inhibitors usually result in induction of the HSR leading to HSP70 overexpression mediated by heat shock factor 1 (HSF1) [50]. HSP70 upregulation has thus been related to the confirmation of HSP90 inhibition [51,52]. Besides HSP70, HSP90 overexpression occurs after HSP90 inhibition due to the HSR after inhibition as well as dissociation of HSF1 from HSP90 [53]. In this way, we have identified compensatory responses specific for each HSP90 inhibitor used, alone or in combination with a HSP70 inhibitor. On the other hand, we found that HSP60 was down-expressed in the EGFR mutant cell line after treatment with STA-9090 and 17-AAG plus VER-155008 combination; however, it was overexpressed in the EML4-ALK cell line in response to STA-9090 inhibition. This protein could be related to STA-9090 sensitivity, and if its inhibition in the EML4-ALK cell line was successful, this could indicate that the HSR may not be as important here as in other cell lines. A cytoprotective role of mitochondrial HSP60 in the apoptotic process was previously described [54,55]. Concretely in lung cancer, low expression of this chaperone has been proposed as a good prognostic factor of disease-free survival [56,57]. HSC71 was detected to be down-regulated after treatment with geldanamycin and radicicol derivatives, which could be related to an optimal response in the EGFR-mutated cell line. However, the expression of this chaperone was increased in response to STA-9090 treatment in the EML4-ALK cell line as well as in the triple negative cell line following IPI-504 treatment. This result is consistent with other studies on different tumor types where HSC71 plays a regulatory role in the HSR and homeostasis [47,58]. Another HSP70 family member, inducible HSPA6, was overexpressed in the H3122 and A549 cell lines following the combined inhibition of HSP90 plus HSP70. The upregulation of this protein, which is only minimally expressed under normal conditions, could be induced through the HSE/HSF1 system [59] and is related to cell survival in response to extracellular stress such as toxicity exposure [60,61]. However, in a recent study, HSPA6 expression was correlated with the inhibition of proliferation, migration and invasion, thereby enhancing the anti-tumor effect of garlic extract in bladder cancer [62]. This could be the reason why we observed a greater number of deregulated pathways in the combined inhibition protocol. Other member of the HSP70 family, mitHSP70, also known as mortalin, has been shown to promote and contribute to the process of carcinogenesis in numerous ways, including the merging and inactivation of tumor suppressor protein p53, which plays a key role in anti-apoptosis pathways, in regulation of the RAS RAF MEK pathway and activation of EMT signalling [63,64]. As mortalin is enriched in many types of cancers its overexpression has been used as a predictor of poor outcome in lung cancer [65,66]. In the present study, mortalin was down-expressed in the EGFR cell line after treatment with the inhibitor 17-AAG and overexpressed in the EML4-ALK cell line with STA-9090. In cancer cells, mortalin responds to stress, including chemicals, and sequesters p53 in the cytoplasm to thereby avoid apoptosis [67]. Since cell death induced by inhibition of HSP90 partly depends on the p53 pathway [33], the mutational status of the tumor suppressor could be in charge of the differential response since in our study the EGFR cell line (HCC827) expresses wild-type (wt) p53 [68] while the EML4-ALK cell line (H3122) expresses mutated p53 [69]. Since wt and mutant p53 as well as Akt, whose action suppresses p53 activity, have been described as HSP90 clients, the inhibition of this chaperone would drive the activation of wt p53 or down-regulation of mutant p53 [70,71]. This fact could be the cause of differential expression in the HCC827 and H3122 cell lines, with degradation of mortalin after nuclear translocation of wt p53 in the EGFR cell line and mortalin accumulation after breaking of the HSP90-mutated p53 complex in the EML4-ALK cell line. Furthermore, the different mutational state of p53 could be related to a larger HSP response in the EML4-ALK cell line than in the EGFR cell line as it was seen with increased HSP90α, HSP60 and HSC71 expression, which could modulate the response to inhibition [72]. In this way, in lung adenocarcinoma cells presenting mutated p53, such as H3122 cells, sensitivity to HSP90 inhibition could be increased by the restoration of wt p53.

Other response biomarkers to HSP90 inhibition were eIF3i, Rab-37, MLH1, AGR2 or transketolase. Deregulation of eIF3, an extremely complex multiprotein assembly with a key role in translation initiation and termination as in ribosomal recycling [73], has been correlated with the onset and progression of cancer [74,75,76]. Concretely, the I subunit of the eIF3 complex (eIF3i) was found to be overexpressed in different tumor types including colon adenocarcinoma and adenoma, head and neck squamous cell carcinomas, hepatocellular carcinoma, breast cancer, cervical cancer and metastatic melanoma [77,78,79,80,81]. Consistent with this, it was reported that eIF3i overexpression leads to malignant phenotype of cells [82,83] and could promote tumor angiogenesis [84,85]. Therefore, the down-expression of eIF3i observed in HCC827 and H3122 cell lines after treatment with HSP90 inhibitors could be a good prognostic factor for this therapeutic strategy in lung adenocarcinoma. Rab-37 is a small Rab-GTPase which regulates both exocytic and endocytic pathways and signal transduction pathways involving cell proliferation, migration, nutrition, innate immunity and fragmentation of compartments [86,87,88]. In lung cancer, low hRAB-37 mRNA expression has been associated with tumor metastasis [89]. It was demonstrated that Rab-37 suppresses lung cancer metastasis by mediating TIMP1 (tissue inhibitor of metalloproteinase) exocytosis, which inactivates extracellular matrix metallopeptidase 9 (MMP9) and suppresses cell invasion signalling [90,91]. However, MMP9 is an HSP90 client, so this protein should be degraded in response to HSP90 inhibition independently of Rab-37. For this reason, expression of this GTPase differed in response to treatments used on the different cell lines studied. However, while the MMP9 was not detected, it should be considered in future experiments for validation of its use as a possible prognostic factor of the response to inhibition. On the other hand, in the study of responses according to inhibitor family, approximately 50% of the differentially expressed proteins were common to inhibitors of the same family. In addition to previously mentioned proteins such as HSP70 and HSP90α, other interesting proteins such as MLH1 and AGR-2 were detected for both HSP90-inhibitor families studied. MLH1 is one of the most important members of the DNA mismatch repair (MMR) system [92], and hence its downregulation was related to tumor progression and chemoresistance in different cancer types, among which is lung cancer [93,94,95,96,97]. In our study, the EGFR cell line showed increased MLH1 expression following treatment with all HSP90 inhibitors. We suggest that inhibition in this context could be restoring the expression of this tumor suppressor and consequently help drug resistance tumors become sensitive to other therapies. For its part, AGR2, which belongs to the protein disulfide isomerase (PDI) family, acts as a chaperone in periods of physiological stress [98]. This protein has been detected to be highly expressed in different tumor types, thus giving rise to the hypothesis that AGR2 acts as an oncogene due to its important role in the activation of survival and metastasis pathways [99,100]. However, other studies showed a down-expression of AGR2 in different cancer types [101,102,103], which raises serious doubts about its oncogenic role. In our study, AGR2 was overexpressed in the EML4-ALK cell line (which has mutated p53) in response to all inhibitor treatments. These findings suggest that AGR2 could serve as a good prognostic factor of HSP90 inhibition, which is in agreement with a recent report where tumor aggressiveness and poorer disease-free survival were related to the downregulation of AGR2, p21 and cyclin D1 in the presence of mutated p53 in ovarian carcinoma [104].

Functional annotation analysis in our study showed that the differentially expressed proteins were involved in 40 different pathways. Six of these pathways (PPP, apoptosis, pyruvate metabolism, glycolysis, 5-HT degradation and ATP synthesis) were shared between all studied HSP inhibitors. In connection with this, the elevated expression of transketolase, an enzyme in the PPP, has been detected in lung cancer [105], confirming an increase in metabolic activity in this tumor type. We found that transketolase was deregulated in all of tested HSP90 inhibitors. However, it was only down-expressed in H3122 cells after inhibition with IPI-504 while it was overexpressed in the A549 and H1437 cell lines under all treatments. These findings suggest that transketolase could have a key role in metabolic activity of lung cancer as well as its downregulation could be also a good prognostic factor.

These results showed a strong intervention of HSP90 inhibition on cell metabolism. Keeping in mind that a hallmark of lung cancer metabolism is the hyperactivity of glycolysis independent of oxygen availability [106,107], we looked for proteins related to this metabolic pathway in our assay. Furthermore, the study of protein expression in cell lines in response to treatment with specific inhibitor families led to the identification of six deregulated pathways following treatment with geldanamycin derivatives and 11 pathways after treatment with radicicol derivatives. Only three of these pathways (glycolysis, PPP and pyruvate metabolism) were shared between families, thus confirming our previous results of the importance of metabolism in HSP90 inhibition. One of the three geldanamycin-specific pathways was the TCA cycle. We showed here that CS, a key enzyme in the TCA cycle, was overexpressed in the KRAS-mutant and triple-negative cell lines after inhibition with 17-AAG and IPI-504. This deregulation could be related to a possible resistance mechanism as was previously reported in ovarian carcinoma where, after chemotherapy, an increase of CS expression levels was related to protection from apoptosis [108,109]. Concerning the 11 specific pathways detected with radicicol derivative treatment, the biosynthesis pathway for serine and glycine was among the most remarkable, since this metabolic pathway, besides leading to the production of amino acids, purines and pyrimidines, is linked to the NADH production needed to maintain redox balance [110]. The upregulation of serine and glycine synthesis has thus been associated with different tumor types [111,112], including lung cancer, where expression of the SHMT1 enzyme has been correlated with good prognosis and expression of SMHT2 with poor prognosis [113]. Interestingly, we found that SMHT2 was downregulated after treatment with STA-9090 in the EGFR cell line, which could be a good signal of treatment efficacy.

In summary, a classical proteomic approach was carried out to detect proteomic changes after HSP90 inhibition in lung adenocarcinoma cell lines. The protein profiles, which were based on 2-DE and mass spectrometry, were identified in response to five different inhibitors. Taken together, we identified a panel of 254 deregulated proteins after HSP90 inhibition. Different proteins detected in our study, such as eIF3i, may provide useful information concerning the specific mode of action of inhibitors. Bioinformatic analyses of the altered proteins suggested that HSP90 inhibitors could affect many pathways, most of which are related to energy production, metabolism and apoptosis. Our results showed that HSP90 interacts with many intracellular pathways which were deregulated after its inhibition. The effects of some of the identified proteins to alter these pathways, such as CS in the TCA cycle, could be related to a possible mechanism that gives rise to an undesired form of HSP90 inhibition. However, further research including models that take tumor microenvironment into account, such as mouse models, as well as clinical investigation, are required to provide context to the significance of these possible biomarkers of response to HSP90 inhibition. Despite the limitations in the models used in this study, the differential protein profiles presented here, such as the pathways identified, contribute to our expanding knowledge of the mechanism of action underlying HSP90 inhibition and the potential development of novel response biomarkers or therapeutic targets.

## 5. Conclusions

We analyzed the proteomics associated with HSP90 inhibition in lung adenocarcinoma using a combined approach of two-dimensional electrophoresis and mass spectrometry analysis. A total of 254 deregulated proteins were found after treatment with HSP90 inhibitors. Functional annotation analysis of differentially expressed proteins revealed different pathways as serine and glycine synthesis or TCA cycle which could help improve our understanding of the biology behind HSP90 inhibition. eIF3i, HSC71, MHC class I antigen, transketolase, citrate synthase, Rab-37, MLH1 and AGR2 might be putative biomarkers in HSP90 inhibition response in lung adenocarcinoma. However, a thorough in vivo validation is required to future clinical uses in lung adenocarcinoma.

## Figures and Tables

**Table 1:** IC50 values of different HSP90 and HSP70 inhibitors in the studied cell lines.

Cell Line	HSP90 Inhibitors				HSP70 Inhibitor	EGFR	ALK	KRAS
	IC50 17-AAG nM	IC50 IPI-504 nM	IC50 STA-9090 nM	IC50 AUY-922 nM	IC50 VER-155008 µM			
**HCC827**	**26.255**	**17.145**	5.138	4.167	2.081	E746-A750 del	WT	WT
**H3122**	26.165	28.371	7.991	9.11	33.898	WT	EML4-ALK v1	WT
**A549**	16.296	19.492	6.31	30.733	24.487	WT	WT	p.G12S
**H1437**	3.708	3.473	6.794	2.814	24.811	WT	WT	WT

nM = nanomolar; µM = micromolar.

**Table 2:** Number of matched protein spots in the considered cell lines under the different treatment conditions.

Condition	Cell Line			
	HCC827	H3122	A549	H1437
**Control**	492	199	263	253
**17-AAG**	488	200	259	264
**IPI-504**	480	202	266	262
**STA-9090**	499	200	235	265
**AUY-922**	507	197	259	261
**VER-155008+17AAG**	374	209	257	275

**Table 3:** Number of differentially expressed proteins identified in the studied cell lines for the different treatments.

Treatment	Protein Deregulation	Cell Line			
		HCC827	H3122	A549	H1437
**17-AAG**	Up	23	4	9	10
	Down	15	3	2	3
**IPI-504**	Up	26	3	11	18
	Down	16	2	6	3
**STA-9090**	Up	25	14	6	9
	Down	18	4	32	3
**AUY-922**	Up	23	5	6	9
	Down	8	4	11	5
**VER-155008 + 17AAG**	Up	30	10	12	15
	Down	61	6	15	3

## Supplementary Materials

The following are available online at https://www.mdpi.com/2073-4409/8/8/806/s1, **Figure S1**: Graphical summary of densitometry results obtained from the Western blots of (**A**) pEGFR, (**B**) EGFR, (**C**) EML4-ALK, (**D**) GRP94, (**E**) HSP90α, (**F**) HSP90β, (**G**) HSP70 and (**H**) HSP27 of Figure S1. All experiments were independently reproduced three times in the laboratory.; **Figure S2:** Graphical summary of densitometry results obtained from the Western blots of (**A**) EGFR, (**B**) HSP90α and (**C**) HSP70 of the cell line HCC827, Western blots of (**D**) EML4-ALK, (**E**) HSP90α and (**F**) HSP70 of the cell line H3122, Western blots of (**G**) EGFR, (**H**) HSP90α and (**I**) HSP70 of the cell line A549, and Western blots of (**J**) HSP90α and (**K**) HSP70 of the cell line H1437; such as Western blots of (**L**) EGFR, (**M**) HSP90α, (**N**) HSP70 after HSP70 and HSP90 inhibition in the cell line HCC827. All of quantified Western blots may be observed in the Figure S2. VER = VER-1555008.; **Figure S3:** Representative two-dimensional (2D) gel images for the cell lines (**A**) HCC827, (**B**) H3122, (**C**) A549 and (**D**) H1437 untreated versus treated with the HSP90 inhibitor 17-AAG. For each cell line, the two spots that were most up- and down-expressed proteins are highlighted by circles, with the accompanying numbers matching those listed in Table S1.; **Figure S4:** Representative two-dimensional (2D) gel images for the cell lines (**A**) HCC827, (**B**) H3122, (**C**) A549 and (**D**) H1437 untreated versus treated with the HSP90 inhibitor IPI-504. For each cell line, the two spots that were most up- and down-expressed proteins are highlighted by circles, with the accompanying numbers matching those listed in Table S2.; **Figure S5:** Representative two-dimensional (2D) gel images for the cell lines (**A**) HCC827, (**B**) H3122, (**C**) A549 and (**D**) H1437 untreated versus treated with the HSP90 inhibitor STA-9090. For each cell line, the two spots that were most up- and down-expressed proteins are highlighted by circles, with the accompanying numbers matching those listed in Table S3.; Figure S6: Representative two-dimensional (2D) gel images for the cell lines (**A**) HCC827, (**B**) H3122, (**C**) A549 and (**D**) H1437 untreated versus treated with the HSP90 inhibitor AUY-922. For each cell line, the two spots that were most up- and down-expressed proteins are highlighted by circles, with the accompanying numbers matching those listed in Table S4.; Figure S7: Representative two-dimensional (2D) gel images for the cell lines (A) HCC827, (**B**) H3122, (**C**) A549 and (**D**) H1437 untreated versus treated with the combination of the HSP70 inhibitor VER-155008 plus the HSP90 inhibitor 17-AAG. For each cell line, the two spots that were most up- and down-expressed proteins are highlighted by circles, with the accompanying numbers matching those listed in Table S5.; **Figure S8:** Graphical summary of densitometry results obtained from the Western blots of the eukaryotic translation initiation factor3 subunit I (eIF3) in the cell lines (**A**) HCC827, (**B**) H3122, (**C**) A549 and (**D**) H1437, such as Western blots of citrate synthase (CS) in the cell lines (**E**) HCC827, (**F**) H3122, (**G**) A549, (**H**) H1437 of Figure 5. All experiments were independently reproduced three times in the laboratory. VER= VER-1555008.; **Table S1:** Differentially expressed proteins identified by mass spectrometry after 17-AAG treatment of the HCC827, H3122, A549 and H1437 cell lines.; **Table S2:** Differentially expressed proteins identified by mass spectrometry after IPI-504 treatment of the HCC827, H3122, A549 and H1437 cell lines.; **Table S3:** Differentially expressed proteins identified by mass spectrometry after STA-9090 treatment of the HCC827, H3122, A549 and H1437 cell lines.; **Table S4:** Differentially expressed proteins identified by mass spectrometry after AUY-922 treatment of the HCC827, H3122, A549 and H1437 cell lines.; **Table S5**: Differentially expressed proteins identified by mass spectrometry after combined treatment with VER-155008 and 17-AAG of the HCC827, H3122, A549 and H1437 cell lines.; **Table S6:** List of 32 shared differentially expressed proteins under geldanamycin derivatives inhibitors.; **Table S7:** List of 52 common deregulated proteins after treatments with radicicol derivatives.
