# Supplementary material for: Impact of Heat Shock Protein 90 Inhibition on the Proteomic Profile of Lung Adenocarcinoma as Measured by Two-Dimensional Electrophoresis Coupled with Mass Spectrometry
Source: Cells. 2019 Jul 31;8(8):806. doi: 10.3390/cells8080806 (PMC6721529; doi:10.3390/cells8080806)
Supplement: Supplementary file 1 [file cells-08-00806-s001.zip › Supplementary table 6.docx]

Supplementary Table 6: List of 32 shared differentially expressed proteins under geldanamycin derivatives inhibitors.

| Common elements in 17AAG and IPI504 : |
| --- |
| 3-hydroxyacyl-CoA dehydrogenase type-2 |
| Alternative protein EML5 |
| Anamorsin |
| Anterior gradient protein 2 homolog |
| C2ORF3 variant 2 |
| Calcium-independent phospholipase A2-gamma |
| Citrate synthase |
| Clathrin coat assembly protein AP180 |
| DNA ligase 3 |
| Elongation factor 2 |
| Enolase |
| HCG1771495, isoform CRA_b |
| Heat shock 70 kDa protein 1A/1B |
| Heat shock 70kDa protein 1A variant |
| Heat shock cognate 71 kDa protein |
| HSP90AA1 protein |
| MHC class I antigen |
| Mitochondrial aldehyde dehydrogenase 2 variant |
| Mitochondrial thiamine pyrophosphate carrier |
| MutL-like 1 protein |
| Phenylalanine--tRNA ligase beta subunit |
| Proliferation-associated protein 2G4 |
| Protein FAM210B |
| RRP12-like protein |
| Serine/threonine-protein phosphatase 2A 56 kDa regulatory subunit gamma isoform |
| SMT3 suppressor of mif two 3 homolog 3 (Yeast), isoform CRA_d |
| SRC kinase-signaling inhibitor 1 |
| Transcription intermediary factor 1-beta |
| Transketolase |
| UDP-glucose 6-dehydrogenase |
| Villin 1 variant |
| V-type proton ATPase 116 kDa subunit a isoform 1 |
