# Supplementary material for: Impact of Heat Shock Protein 90 Inhibition on the Proteomic Profile of Lung Adenocarcinoma as Measured by Two-Dimensional Electrophoresis Coupled with Mass Spectrometry
Source: Cells. 2019 Jul 31;8(8):806. doi: 10.3390/cells8080806 (PMC6721529; doi:10.3390/cells8080806)
Supplement: Supplementary file 1 [file cells-08-00806-s001.zip › Supplementary table 7.docx]

Supplementary Table 7: List of 52 common deregulated proteins after treatments with radicicol derivatives.

| Common elements in STA9090 and AUY922 : |
| --- |
| 3-hydroxyacyl-CoA dehydrogenase type-2 |
| Acyl-coenzyme A thioesterase 8 |
| Adenosine deaminase, RNA-specific, isoform CRA_b |
| Alternative protein EML5 |
| Alternative protein FGF1 |
| Alternative protein ILF3 |
| Anterior gradient protein 2 homolog |
| ATP synthase subunit beta |
| B-Raf protein |
| C2ORF3 variant 2 |
| Calcium-independent phospholipase A2-gamma |
| Coiled-coil domain-containing protein 90B, mitochondrial |
| DNA ligase 3 |
| E3 ubiquitin-protein ligase ZNRF2 |
| Elongation factor 2 |
| Enolase |
| Eukaryotic translation initiation factor 3 subunit E |
| Eukaryotic translation initiation factor 3 subunit I |
| Filamin-binding LIM protein 1 |
| Glucokinase |
| Glucose-6-phosphate 1-dehydrogenase |
| HCG1771495, isoform CRA_b |
| HCG2022736, isoform CRA_a |
| Heat shock 70 kDa protein 1A/1B |
| Heat shock 70kDa protein 1A variant |
| Heat shock 70kDa protein 8 isoform 1 variant |
| HSP90AA1 protein |
| MHC class I antigen |
| Mitochondrial aldehyde dehydrogenase 2 variant |
| MutL-like 1 protein |
| NAD(P)H dehydrogenase [quinone] 1 |
| Phenylalanine--tRNA ligase beta subunit |
| Phosphoserine aminotransferase |
| Poly [ADP-ribose] polymerase 2 |
| Protease serine 1 |
| Protein FAM210B |
| Protein zyg-11 homolog B |
| Pyruvate kinase PKM |
| Radixin |
| RDX protein |
| Serine/threonine-protein phosphatase 2A 56 kDa regulatory subunit gamma isoform |
| Serpin H1 |
| S-formylglutathione hydrolase |
| Small ubiquitin-related modifier 3 |
| Transketolase |
| TTL/TEL fusion protein TTL-B2 |
| Upstream ORF |
| Villin 1 variant |
| Vinculin |
| Vinexin |
| V-type proton ATPase 116 kDa subunit a isoform 1 |
| YWHAE/FAM22A fusion protein |
