# Supplementary figures and images for: Impact of Heat Shock Protein 90 Inhibition on the Proteomic Profile of Lung Adenocarcinoma as Measured by Two-Dimensional Electrophoresis Coupled with Mass Spectrometry
Source: Cells. 2019 Jul 31;8(8):806. doi: 10.3390/cells8080806 (PMC6721529; doi:10.3390/cells8080806)

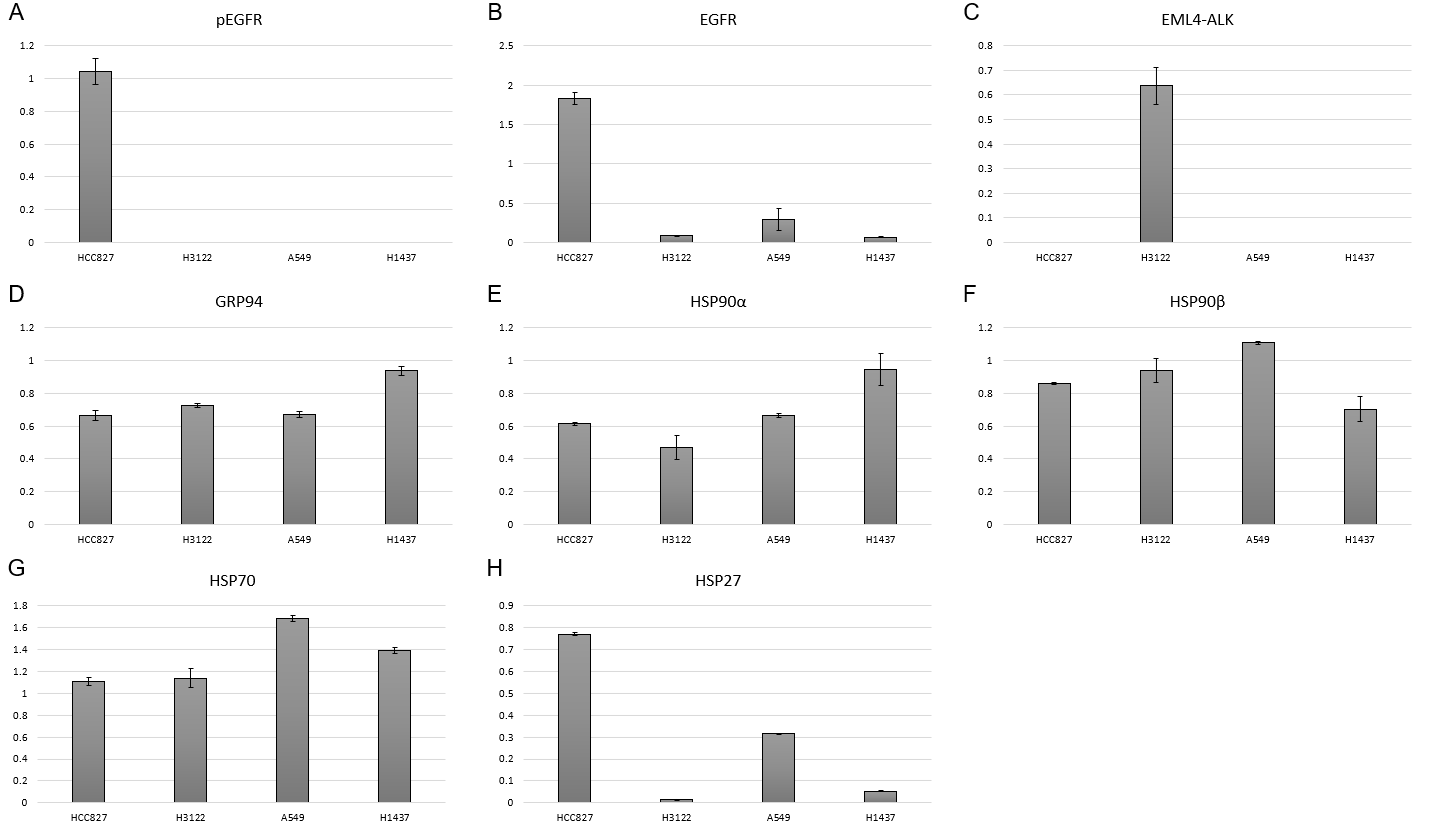

Supplement: Supplementary file 1 [file cells-08-00806-s001.zip › Figure S1.png]

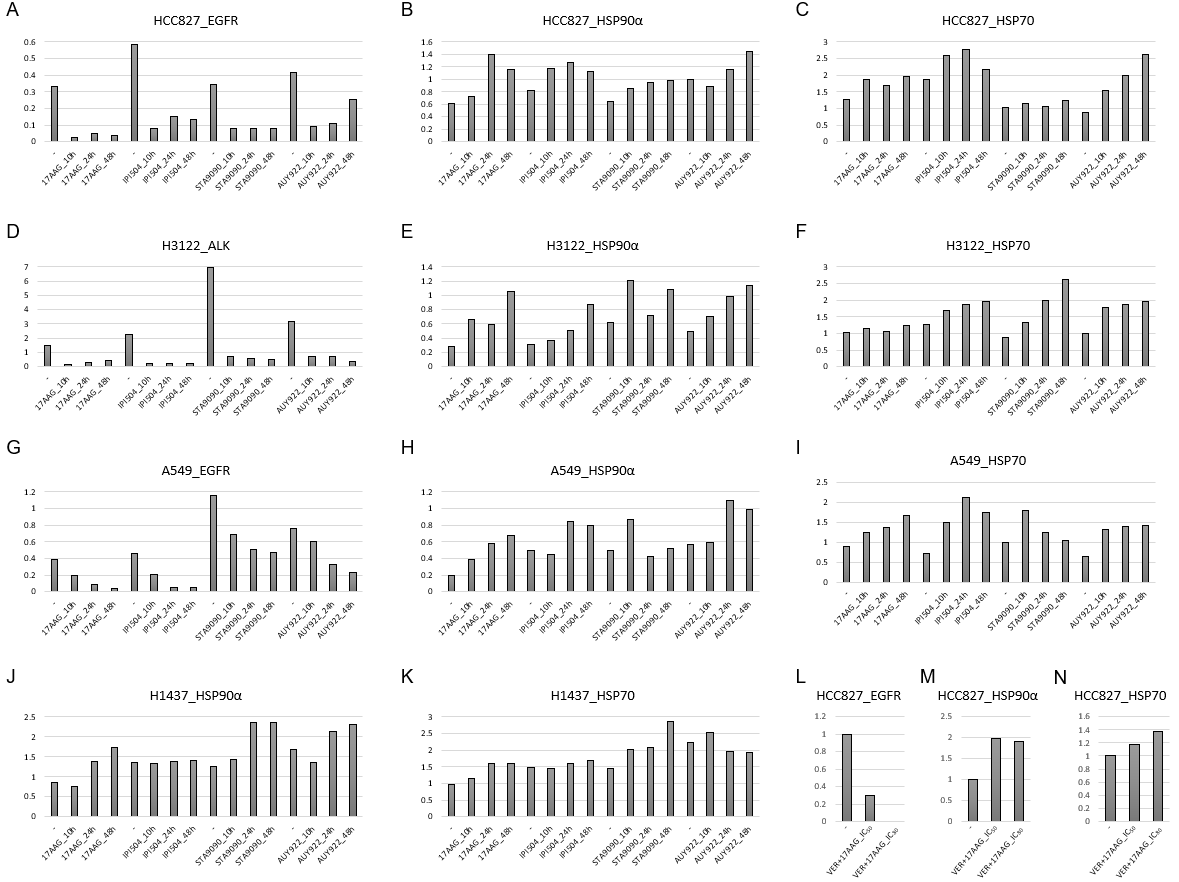

Supplement: Supplementary file 1 [file cells-08-00806-s001.zip › Figure S2.png]

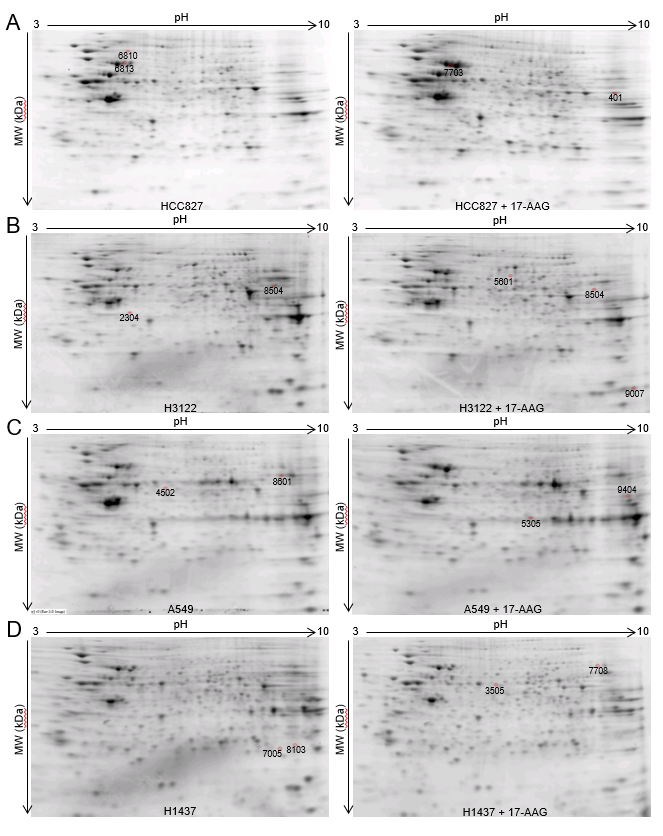

Supplement: Supplementary file 1 [file cells-08-00806-s001.zip › Figure S3.png]

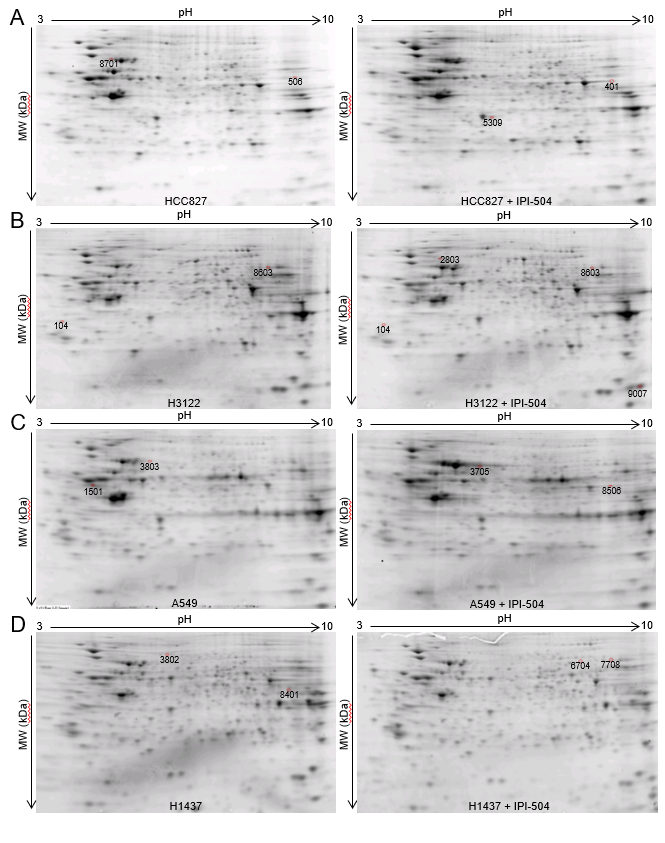

Supplement: Supplementary file 1 [file cells-08-00806-s001.zip › Figure S4.png]

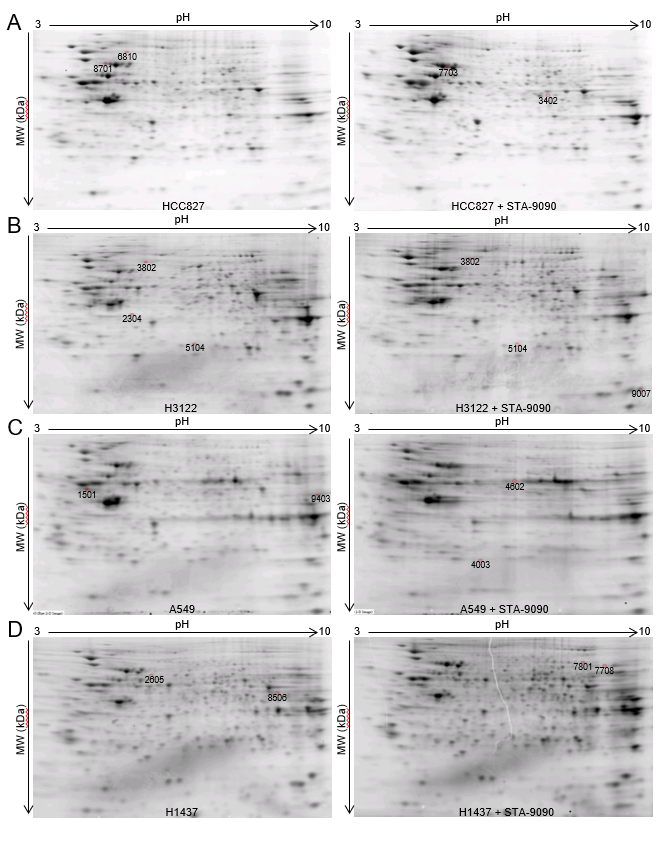

Supplement: Supplementary file 1 [file cells-08-00806-s001.zip › Figure S5.png]

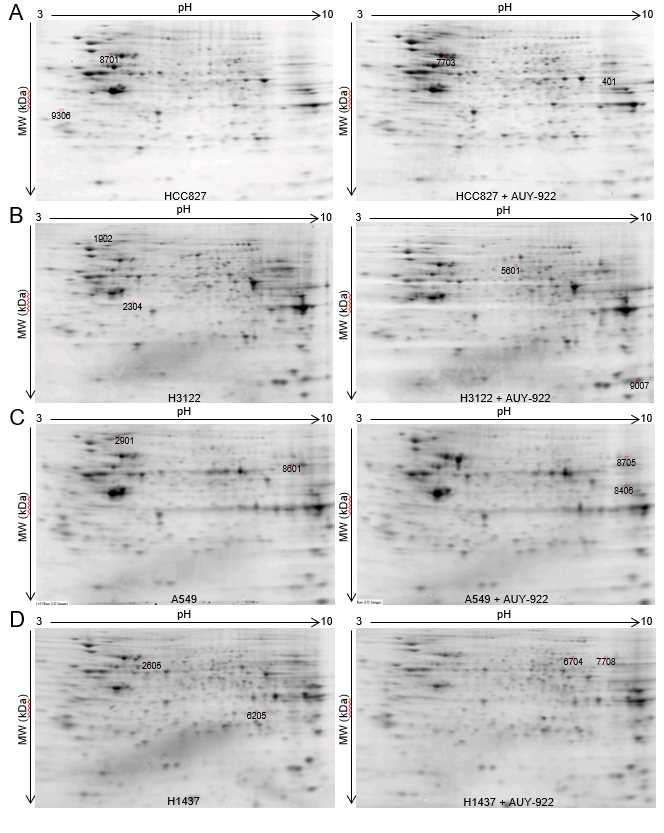

Supplement: Supplementary file 1 [file cells-08-00806-s001.zip › Figure S6.png]

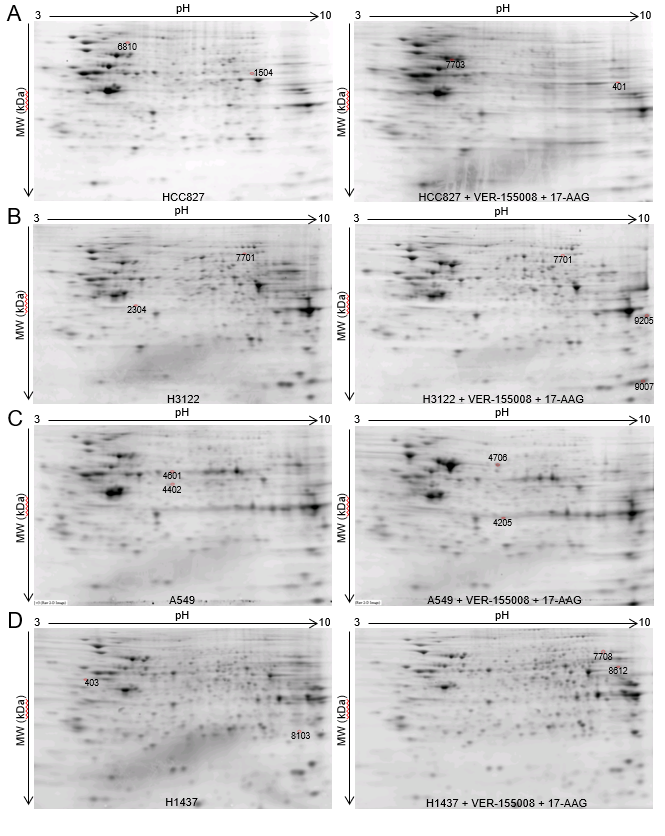

Supplement: Supplementary file 1 [file cells-08-00806-s001.zip › Figure S7.png]

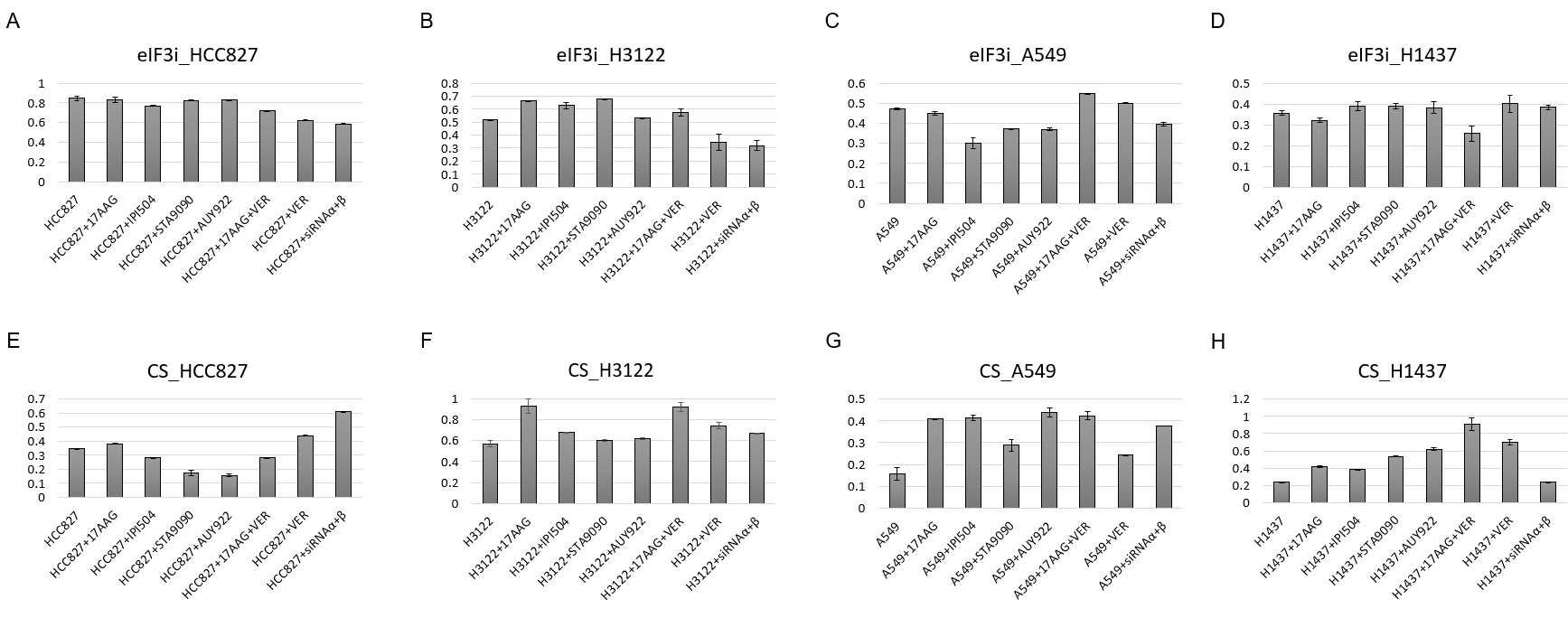

Supplement: Supplementary file 1 [file cells-08-00806-s001.zip › Figure S8.png]
